# Association of mortality and endothelial dysfunction with serum ADMA level in COVID-19 patients

**DOI:** 10.12669/pjms.38.7.5327

**Published:** 2022

**Authors:** Cengiz Karacaer, Selcuk Yaylaci, Taner Demirci, Deniz Cekic, Kezban Ozmen Suner, Erdem Cokluk, Ceyhun Varim

**Affiliations:** 1 Cengiz Karacaer, Medical Doctor, Department of Internal Medicine, Sakarya University Research and Education Hospital, Sakarya, Turkey; 2 Selcuk Yaylaci, Associate Professor, Department of Internal Medicine, Sakarya University Research and Education Hospital, Sakarya, Turkey; 3 Taner Demirci, Assistant Professor, Department of Endocrinology and Metabolism, Sakarya University Research and Education Hospital, Sakarya, Turkey; 4 Deniz Cekic, Medical Doctor, Department of Internal Medicine, Sakarya University Research and Education Hospital, Sakarya, Turkey; 5 Kezban Ozmen Suner, Assistant Professor, Department of Reanimation and Anesthesiology. Sakarya University Research and Education Hospital, Sakarya, Turkey; 6 Erdem Cokluk, Assistant Professor, Department of Biochemistry. Sakarya University Research and Education Hospital, Sakarya, Turkey; 7 CeyhunVarim: Associate Professor, Department of Internal Medicine, Sakarya University Research and Education Hospital, Sakarya, Turkey

**Keywords:** Asymmetric dimethylarginine, COVID-19, Mortality rate, Endothelial dysfunction, Lymphocyte to albumin ratio

## Abstract

**Objectives:**

To investigate the role of asymmetric dimethylarginine (ADMA) level in predicting intensive care and mortality in patients affected with coronavirus disease 2019 (COVID-19).

**Methods:**

This retrospective, cross-sectional study was conducted at Sakarya University Training and Research Hospital (Sakarya, Turkey) between April and August of 2020. We enrolled patients who were diagnosed with COVID-19 via real-time reverse-transcription polymerase chain reaction and admitted to the intensive care (Severe COVID-19; S-COVID) or non intensive care (Moderate COVID-19; M-COVID). We then analyzed the relationship of the ADMA level with various parameters between S-COVID and M-COVID groups.

**Results:**

This study included 87 patients, comprising 43 females and 44 males, with a mean age of 61 and 71.50 years, respectively. The male/female distribution was 22/25 (46.8%/53.2%) in the M-COVID group and 22/18 (55%/45%) in the S-COVID group. The hospitalization time, white blood cell count, neutrophil count, lymphocyte-to-albumin ratio, international normalization ratio, D-dimer, troponin, ferritin, lactate dehydrogenase, C-reactive protein, procalcitonin, erythrocyte sedimentation rate, fibrinogen, lactate, ADMA, and mortality rate were significantly higher (p < 0.05). In contrast, lymphocyte, total cholesterol, high-density lipoprotein, calcium, and albumin values were lower (p < 0.05) in the S-COVID group than in the M-COVID group. While the mortality rate was 55% in S-COVID patients, no mortality was detected in M-COVID patients (p < 0.05). Moreover, ADMA level was 6618 ± 3000 (6400) in S-COVID patients and 5365 ± 3571 (3130) in M-COVID patients, indicating a statistically significant difference (p = 0.012).

**Conclusion:**

The asymmetric dimethylarginine level increases in severe outcomes; hence, it can potentially predict severity in patients with COVID-19.

## INTRODUCTION

The pathological specimens of a patient severely affected with coronavirus disease 2019 (COVID-19) have been examined; the virus invades the endothelial cells of glomerular capillaries, causing endotheliitis in multiple regions including the heart, lung, kidney, liver, and gastrointestinal tract.^1^ The impact of the virus on the endothelium caused secondary myocardial inflammation and dysfunction, leading to significant effects on vascular homeostasis and the coagulation system by the vascular endothelial cells.^2^,^3^

The relative importance of endothelial dysfunction (ED) in viral infections is very well known. Although less contagious, the H5N1 avian flu strain, which aroused great concern a few years ago, causes significant edema and even death in humans; its high mortality rate is associated with cytokine storm, which disrupts microvascular barrier integrity.^4^ Accordingly, researchers have extensively investigated whether severe acute respiratory syndrome coronavirus 2 (SARS-CoV-2) has the same phenomenon as other influenza strains, including swine flu H1N1 and seasonal influenza strains.^5^

Recently, the signs and symptoms of COVID-19 are reported to be similar to the clinical phenotype of ED, sharing common pathophysiological mechanisms.^6^-^8^ COVID-19 affects the lungs and endothelial system. In various viral infections, including previous coronaviruses, ED has been suggested as the major pathophysiological process. Direct viral infection of the endothelium or the recruitment of immune-mediated immune cells could cause widespread ED associated with apoptosis.^9^

Our literature review confirms previous and recent reports that claim that endothelial dysfunction plays a key role in viral infections. This knowledge is important to understand the multisystemic attacks of these viruses and execute appropriate patient management.^10^ Considering previous experience from other coronaviruses, complement activation has been reported as part of the vicious cycle of ED in COVID-19.^11^

We thought that COVID-19 infection also causes thromboembolic conditions as a result of endothelial damage, and we thought that asymmetrical dimethylarginine (ADMA), which is used as a biomarker in endothelial dysfunction, may be useful in determining the prognosis of COVID-19 disease. However, in the literature review, few study on the ADMA level in COVID-19 has been found. Hence, our study aimed to investigate the role of ADMA level in predicting intensive care and mortality in patients affected with COVID-19.

## METHODS

This retrospective, cross-sectional, and single-centered study enrolled 87 patients admitted to Sakarya University Training and Research Hospital (Sakarya, Turkey) between April and August of 2020. Information including age, gender, chronic diseases, hospitalization-intensive care admission, hospitalization duration, symptoms, and medications were recorded on the automation system within the scope of this study. The patients were categorized into two: intensive care unit (ICU) group (S-COVID) and non intensive care group (M-COVID). The study protocol was approved by the Ethics Committee of Sakarya University Medical Faculty and was conducted by the Declaration of Helsinki (71522473/050.01.04/519).

Heparinized blood gas injectors were used for blood gas analysis, EDTA containing tubes (CBC) for complete blood count, tubes containing 3.8% sodium citrate for coagulation parameters and anticoagulant-free biochemistry tubes for serum tests.

### Inclusion & Exclusion Criteria

Patients with RT-PCR negative, immunosuppressive therapy, pregnancy, and patients under 18 years of age were excluded from the study. Our inclusion criteria are; being over 18 years old, not pregnant. Patients with confirmed COVID-19 diagnosis using polymerase chain reaction (PCR) method and/or computed tomography (CT) were included in the study. Diabetes mellitus was considered as fasting blood glucose concentration > 126 mg/dL or any blood glucose measurement > 200 mg/dL or use of anti-diabetic medication. Hypertension was defined as blood pressure above 130/85 mmHg or use of antihypertensive medication. ADMA kit was paid for by the researchers and was studied in the biochemistry laboratory of our hospital by the author who participated in the research.

The data were retrieved from the hospital information system. For measuring the ADMA levels, patients’ serum were centrifuged, collected, and stored at −80 until the experiments were performed. On the test day, all samples were initially conditioned to room temperature (15–18); then, they were homogenized and measured. ADMA levels were determined using a double antibody enzyme-linked immunosorbent assay (YLBiont brand Sandwich ELISA; Shanghai YL Biotech Co., Ltd., Shanghai, China). Hormone specific monoclonal antibody coated wells. Streptavidin–HRP-conjugated antibodies were added to all wells, except the blank well, and the wells were incubated at 37°C for 60 min. After incubation, the wells were washed to remove unbound antibody. The specimens were incubated with chromogen at 37°C for 10 minutes to develop a blue color. Stop solution was added to terminate the reaction, reflected by a change in the color of the solution from blue to yellow. The intensity of the yellow color was directly proportional to the analyte concentration. The colorimetric readings were performed using the inappropriate wavelength for the micro ELISA reader. A standard curve was generated to calculate the sample concentrations. In the precision study conducted by the manufacturer, the interstudy and intrastudy CV% values of the kits were <10%, and the measurement range was 200–60.000 ng/L.

Biochemical parameters and C-reactive protein (CRP) values were measured by immunoturbidimetric method using Olympus AU5800 autoanalyzer (Beckman Coulter, Inc. Brea, CA92821 USA). The ferritin level was detected by chemiluminescence using Abbott Architect I 2000 SR (Abbott Laboratories Abbott Park IL, 60064, USA). CBC parameters were identified through laser measurements and LED flow cell method using CELL-DYN 3700 CD-3700SL (Abbott Diagnostics Division, Abbott Laboratories Abbott Park IL, 60064, USA). In measuring the prothrombin time, activated partial thromboplastin time, and fibrinogen, an optical method was used. Furthermore, D-dimer was detected through latex agglutination method using DiagonCoagXL (Budapest, Hungary) device, while the erythrocyte sedimentation rate (ESR) was measured using Vacuplus ESR-20 (Turkey) fully automated ESR analyzer. Procalcitonin was measured by the immunoassay method using Roche Cobas e 411 (Hitachi, 6544-01 Tokyo Japan).

### Statistical Analysis

Descriptive statistics are represented as mean values, standard deviation values, median values, minimum and maximum value frequencies, and percentages. The distribution of the variables was evaluated by Kolmogorov–Smirnov test. The quantitative and qualitative data were compared by Mann-Whitney *U*-test and chi-square test, respectively. Additionally, the level of the effect was analyzed by ROC analysis and demonstrated by logistic regression (univariate–multivariate model) (Forward-LR). All statistical analyses were performed using SPSS Statistics 26.0.

## RESULTS

This study enrolled 87 patients, comprising 43 (49.4%) males and 44 (50.6%) females. The male/female distribution was 22/25 (46.8/53.2%) in the M-COVID group and 22/18 (55/45%) in the S-COVID group, with a mean age of 71.50/61 years.

These patients mostly complained of coughing (54%), weakness (50.6%), dyspnea (47.1%), and fever (35.6%). Among the most common comorbidities were hypertension (20.7%), diabetes mellitus type 2 (32.2%), coronary artery disease (20.7%), and chronic obstructive pulmonary disease (11.5%) (Table-I).

**Table I:** Symptoms and comorbidities of all patients.

	Results*(n=87)
Age, years (interquartile range)	64 (54-73)
Gender, F/M (%)	43 / 44 (49.4 / 50.6)
Initial Symptoms	
Cough	54 (47)
Fever	35.6 (31)
Sore throat	11.5 (10)
Dyspnea	47.1 (41)
Weakness	50.6 (44)
Sputum	6.9 (6)
Headache	4.6 (4)
Comorbidities	
Hypertension	20.7(18)
Chronic Obstructive Pulmonary Disease	11.5 (10)
Chronic Renal Failure	10.3 (9)
Diabetes Mellitus Type-2	32.2(28)
Malignancy	2 (2.3)
Coronary Arterial Disease	20.7(18)
Cerebrovascular accident	6.9(6)

Gender distribution was similar in both groups (p > 0.05). The hospitalization time, white blood cell (WBC) count, neutrophil count, international normalization ratio, D-dimer, troponin, ferritin, lactate dehydrogenase (LDH), CRP, procalcitonin, ESR, fibrinogen, lactate, ADMA, and mortality rate were significantly higher in the S-COVID group than in the M-COVID group (p < 0.05) (Table-II).

**Table II:** Comparison of laboratory parameters of intensive care and clinical patients.

		M-COVID Group		S-COVID Group		p	

		Mean±sd/n-%	Median	Mean±sd/n-%	Median		
Age		56.3±16.6	61,0	69.6±14.1	71.5	0.000	^m^
Gender	Female	25±53.2%		18±45.0%		0.446	^X²^
	Male	22 ±46.8%		22±55.0%			
Hospitalization time		34.8±12.4	31,0	65.4±46.2	49.5	0.001	^m^
WBC (x10³)		6.25±1.96	6,00	8.82±4.43	7.42	0.001	^m^
HGB		12.50±1.30	12,70	11.99±1.88	11.95	0.251	^m^
Lymphocyte		1.45±0.79	1,30	0.87±0.53	0.81	0.000	^m^
Neutrophil		4.23±1.89	3,89	6.56±3.27	5.64	0.000	^m^
PLT		187.5±57.5	176,0	234.9±137.5	206.5	0.157	^m^
INR		1.17±0.32	1,07	1.41±0.89	1.23	0.000	^m^
D-Dimer		687.8±940.8	435,0	3443.2±7588.9	1280.0	0.000	^m^
Troponin		17.1±47.4	3,6	74.2±189.6	13.4	0.000	^m^
LDL		95.2±21.4	100,0	89.6±31.5	89.0	0.332	^m^
T. Cholesterol		158.0±32.3	157,0	138.6±38.2	130.0	0.025	^m^
HDL		40.2±10.8	40,0	30.3±7.0	31.0	0.000	^m^
Ferritin		297.5±592.8	119,4	1007.4±1590.3	490.0	0.000	^m^
FPG		136.3±72.2	110,0	138.7±60.5	119.0	0.373	^m^
Creatine		0.78±0.20	0,75	1.09±0.76	0.85	0.060	^m^
Ca		8.85±0.67	9,00	8.45±0.58	8.50	0.000	^m^
Albumin		3.60±0.38	3,60	3.03±0.37	3.10	0.000	^m^
LDH		288.5±97.6	280,0	479.8±176.7	461.0	0.000	^m^
CRP		39.0±45.7	20,0	106.7±57.4	94.0	0.000	^m^
Procalcitonin		0.38±1.23	0,06	1.03±2.77	0.19	0.000	^m^
Sedimentation		37.3±25.0	29,0	62.5±23.4	68.0	0.000	^m^
Fibrinogen		349.1±68.4	326,0	394.0±93.1	394.0	0.011	^m^
CK		155.4±255.7	81,0	253.1±400.4	102.0	0.111	^m^
CKMB		17.0±7.5	14,4	32.9±60.2	18.0	0.197	^m^
Lactate		1.60±0.59	1,65	1.86±0.59	1.90	0.033	^m^
ADMA		5365±3571	3130	6618±3000	6400	0.012	^m^
Mortality, positive		0 ±0.0%		22 ±55.0%		0.000	^X²^
^m^ Mann-Whitney U test/^X²^ Chi-square test							

WBC: White blood cell count, HGB: Hemoglobin, PLT: Platelet, INR: International Normalized Ratio, LDL: Low-density lipoprotein, HDL: High-density lipoprotein, FPG: Fasting Plasma Glucose, Ca: Calcium, LDH: Lactate dehydrogenase, CRP: C-Reactive Protein CK: Creatine kinase, CKMB: Creatine kinase myocardial band, ADMA: Asymmetrical Dimethylarginine.

Moreover, the S-COVID group had significantly lower lymphocyte count, total cholesterol, high-density lipoprotein (HDL), calcium, and albumin than the M-COVID group (p < 0.05). However, no significant difference was detected in hemoglobin, platelet count, low-density lipoprotein, fasting plasma glucose, creatine, creatine kinase, and creatine kinase myocardial band between the two groups (p > 0.05) (Table-II).

In the univariate model, significant efficacies were observed in age, hospital admission time, WBC, lymphocyte, neutrophil, D-dimer, total cholesterol, HDL, ferritin, calcium, albumin, LDH, CRP, sediment, fibrinogen, and lactate values (p < 0.05) (Table-III). Meanwhile, the multivariate model detected significant independent efficiency in lymphocyte and albumin values (p < 0.05) (Table-III).

**Table III:** Evaluation of the univariate-multivariate models of the laboratory parameters and logistic regression analysis.

	Univariate Model			Multivariate Model		
	
	OR	95% Confidence Level	P	OR	95% Confidence Level	p
Age	1.059	1.025-1.095	0.001			
Hospitalization time	1.058	1.011-1.108	0.015			
WBC (x10³)	1.000	1.000-1.000	0.002			
Lymphocyte	0.181	0.068-0.483	0.001	0.023	0.001-0.765	0.035
Neutrophil	1.468	1.176-1.831	0.001			
INR	6.369	0.745-54.416	0.091			
D-Dimer	1.001	1.000-1.002	0.001			
Troponin	1.009	0.999-1.019	0.073			
T. Cholesterol	0.984	0.971-0.998	0.026			
HDL	0.874	0.809-0.943	0.001			
Ferritin	1.001	1.000-1.002	0.020		
Ca	0.312	0.134-0.727	0.007			
Albumin	0.012	0.002-0.089	0.000	0.001	0.0001-0.374	0.022
LDH	1.011	1.006-1.016	0.000			
CRP	1.025	1.014-1.036	0.000			
Procalcitonin	1.190	0.903-1.568	0.217			
Sedimentation	1.042	1.019-1.065	0.000			
Fibrinogen	1.007	1.001-1.013	0.022			
Lactate	2.187	1.006-4.754	0.048			
ADMA	1.000	1.000-1.000	0.086			

WBC: White blood cell count, INR: International Normalized Ratio,, HDL: High-density lipoprotein , LDH: Lactate dehydrogenase, CRP: C-Reactive Protein, ADMA: Asymmetrical Dimethylarginine, Logistic Regression/(Forward-LR)

**Fig.1:**
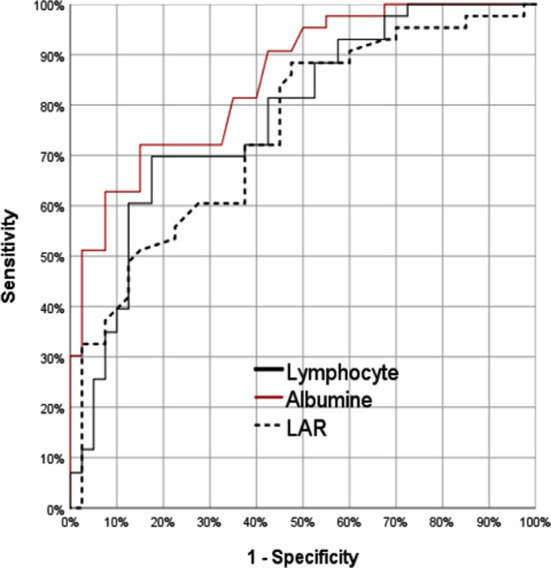
The area under the receiver operating characteristic curves of lymphocyte to albumin ratio, lymphocyte counts and albumin. The plasma ADMA levels of both groups individually is illustrated in Fig-2. While the S-COVID group obtained a mortality rate of 55%, the M-COVID group detected no mortality (p < 0.05).

In the differentiation of the S-COVID and M-COVID groups, the lymphocyte value AUC: 0.778 [0.678-0.878] revealed significant efficacy. At the lymphocyte cutoff value of 1.1, the sensitivity, positive predictive, specificity, and negative predictive were 82.5%, 64.7%, 61.7%, and 80.6%, respectively. Likewise, the albumin value (AUC: 0.851 [0.772-0.930]) demonstrated significant efficacy in the differentiation of both groups, with 85.0% sensitivity, 68.0% positive predictive, 62.8% specificity, and 81.8% negative predictive at the albumin cutoff value of 3.4. Additionally, the differentiation of both groups detected significant efficacy of the lymphocyte to albumin ratio (LAR) value (AUC: 0.851 [0.772–0.930]), with 50.0% sensitivity, 80.0% positive predictive, 89.4% specificity, and 67.7% negative predictive at the LAR cutoff value of 0.62 (Fig.1).

**Fig.2:**
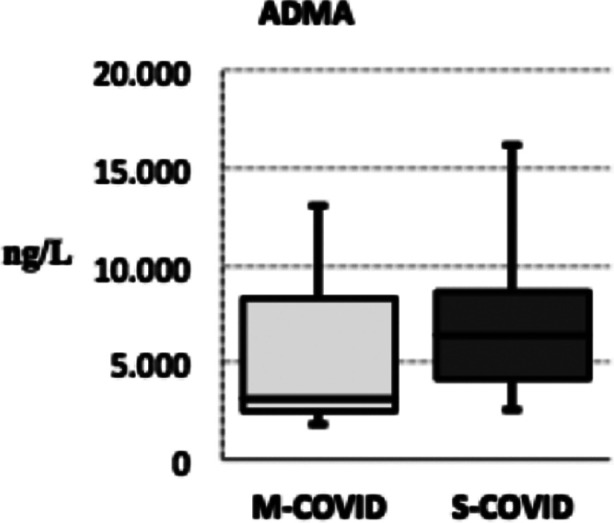
Plasma ADMA levels of the M-COVID and S-COVID groups.

## DISCUSSION

In our study, the ADMA levels and mortality rates of the S-COVID group, which consisted of patients who were treated in the ICU, were higher than those of the M-COVID group or the patient group that did not require intensive care. These differences were statistically significant (p < 0.05). Conversely, the S-COVID group had statistically lower lymphocyte and albumin values than the M-COVID group (p < 0.05) (Table-II). While the efficacies of WBC, lymphocyte, neutrophil, D-dimer, ferritin, albumin, CRP, and sedimentation were significant in the univariate model (p < 0.05), significant independent efficiencies of lymphocyte and albumin values were observed in the multivariate reduced model (p < 0.05) (Table-III). Thus, our data clearly represent a cellular response characterized by elevated ADMA levels, hypoalbuminemia, and lymphocytopenia.

Mahida, RY et al. demonstrated that patients who were infected with SARS-CoV-2 and admitted to the ICU had diminished albumin levels but had elevated CRP levels and platelet counts.^12^ In addition, as the length of ICU stay prolonged, differences in CRP and albumin among patient groups increased; in contrast, no difference in lymphocyte count was detected between such groups. In the study of Guan, WJ et al., patients with severe diseases had abnormal laboratory results such as lymphocytopenia and leukopenia compared with those without severe diseases.^13^

Lymphocytopenia is a prominent feature in critically ill patients with SARS-CoV infection, considering that the targeted invasion of SARS-CoV viral particles severely damages the cytoplasmic component of the lymphocyte. Therefore, lymphocyte necrosis or apoptosis causes lymphocytopenia in critically ill patients with SARS-CoV-2 infection.^14^ In our study, low albumin and lymphocyte levels, along with high CRP and leukocyte levels, were detected in the S-COVID group, similar to the previous literature. Hence, these parameters are considered as prognostic indicators in the literature.

Endothelial cell damage may ED is a systemic condition wherein the endothelium loses its physiological properties, including promotion of vasodilation, fibrinolysis, and antiaggregation.^15^ In this condition, inflammatory cells are accumulated, as evidenced by the occurrence of endotheliitis containing viral elements within the endothelial cells, leading to endothelial and inflammatory cell death. The virus enters the endothelial cells through endocytosis by binding the spike glycoprotein to a cellular receptor that facilitates viral binding to the surface of target cells.^16^,^17^

SARS-CoV-2 infection and the ACE2 receptor effect lead to the activation of endothelial cells, especially in systemic vessels (small and large arteries, veins, venules, and capillaries). Thus, these blood vessels become dysfunctional.^18^ Endothelial activation and ED result in the increase of proinflammatory cytokines such as interleukin-1, interleukin-6 [IL-6], tumor necrosis factor-α (TNF-α), and chemokines such as monocyte chemoattractant protein-1, as well as von Willebrand factor (vWF) antigen, vWF activity, and factor VIII. Furthermore, SARS-CoV-2 infection is associated with high levels of acute phase reactants, including IL-6, CRP, and D-dimer.^19^

Therefore, ED contributes to COVID-19-associated vascular inflammation, especially endotheliitis in the lung, heart, and kidney, as well as COVID-19–associated coagulopathy, particularly microthrombi with pulmonary fibrin in alveolar capillaries. Patients with severe COVID-19 reportedly have high levels of proinflammatory cytokines such as IL-2R, IL-6, and TNF-α. These cytokines play a major role in inducing the loss of normal antithrombotic and anti-inflammatory functions of endothelial cells.^20^,^21^

Numerous studies have concentrated on ADMA in ED disorder and cardiovascular mortality.^21^-^23^ In impaired endothelial function cases, increased ADMA levels have been associated with decreased production of nitric oxide (NO), which is involved in various regulatory mechanisms of the cardiovascular system. Recent studies have declared ADMA as a risk factor of ED. Patients with hypertension and chronic heart failure exhibit high ADMA plasma concentrations.^24^ ED is a contributing factor to vasculopathy and coagulopathy in COVID-19. Various consequences of dysfunction in the context of vascular endothelium and COVID-19 have been considered cardinal features. ED is a key event during the initiation and progression of atherosclerotic vascular disease. It is characterized by reduced NO bioavailability. Endothelial “activation” occurs, which in turn, leads to a proinflammatory, proliferative, and prothrombotic state of the endothelium.

In a study by Hannemann et al., it was suggested that increased serum ADMA level could be used as a new biomarker to determine mortality in COVID-19 patients.^25^ In our study, serum ADMA levels were statistically significantly higher in the S-COVID patient group than in the M-COVID patient group (p = 0.012)

### Limitations of the study

It is single-center, has a relatively small number of patients. It requires more studies with larger patient numbers and groups.

In present study, the ADMA level was significantly higher in patients with COVID-19 treated in the ICU (S-COVID). To our knowledge, the ADMA levels in patients affected with COVID-19 have remained uninvestigated. Hence, we believe that our study will contribute to the current literature.

## CONCLUSION

The ADMA levels increase as the disease worsens, suggesting that ADMA can indicate ED. Hence, it can be used as a marker of severity in patients suffering from COVID-19.

### Authors’ Contribution:

**CK:** Conceived the study, literature review, participated in its design, coordination, analyzed the data and helped to draft the manuscript and also the responsible and accountable for the accuracy or integrity of the work.

**SY:** Contributed to data collecting .

**TD:** Contributed to the conception of the work, revising the draft, approval of the final version of the manuscript, and agreed for all aspects of the work.

**DC:** Takes the responsibility and is accountable for all aspects of the work in ensuring that questions

related to the accuracy and integrity of any part of the work are appropriately investigated and resolved.

**KOS & EC:** Helped in design, data collection, drafting the manuscript & critical revision.

**CV:** Contributed to the conception of the work, revising the draft.
